# Mediating oxidative stress through the Palbociclib/miR-141-3p/STAT4 axis in osteoporosis: a bioinformatics and experimental validation study

**DOI:** 10.1038/s41598-023-46813-6

**Published:** 2023-11-10

**Authors:** Jiajia Ji, Shaobo Wu, Xueyuan Bao, Shixuan Liu, Yuxing Ye, Jiayuan Liu, Jinniu Guo, Jiateng Liu, Xi Wang, Zhihao Xia, Liangliang Wei, Yan Zhang, Dingjun Hao, Dageng Huang

**Affiliations:** 1 Department of Spine Surgery, Honghui Hospital, Xi’an Jiaotong University, Xi’an, 710054 Shaanxi China; 2 Department of Infectious Diseases and Public Health, Jockey Club College of Veterinary Medicine and Life Sciences, City University of Hong Kong, Hong Kong, China; 3 Center for Translational Medicine, The First Affiliated Hospital of Xi’an Jiaotong University, Xi’an, 710061 Shaanxi China

**Keywords:** Computational biology and bioinformatics, Drug discovery, Genetics

## Abstract

Osteoporosis is a common bone disease characterized by loss of bone mass, reduced bone strength, and deterioration of bone microstructure. ROS-induced oxidative stress plays an important role in osteoporosis. However, the biomarkers and molecular mechanisms of oxidative stress are still unclear. We obtained the datasets from the Gene Expression Omnibus (GEO) database, and performed differential analysis, Venn analysis, and weighted correlation network analysis (WGCNA) analysis out the hub genes. Then, the correlation between inflammatory factors and hub genes was analyzed, and a Mendelian randomization (MR) analysis was performed on cytokines and osteoporosis outcomes. In addition, “CIBERSORT” was used to analyze the infiltration of immune cells and single-cell RNA-seq data was used to analyze the expression distribution of hub genes and cell–cell communications. Finally, we collected human blood samples for RT-qPCR and Elisa experiments, the miRNA-mRNA network was constructed using the miRBase database, the 3D structure was predicted using the RNAfold, Vfold3D database, and the drug sensitivity analysis was performed using the RNAactDrug database. We obtained three differentially expressed genes associated with oxidative stress: DBH, TAF15, and STAT4 by differential, WGCNA clustering, and Venn screening analyses, and further analyzed the correlation of these 3 genes with inflammatory factors and immune cell infiltration and found that STAT4 was significantly and positively correlated with IL-2. Single-cell data analysis showed that the STAT4 gene was highly expressed mainly in dendritic cells and monocytes. In addition, the results of RT-qPCR and Elisa experiments verified that the expression of STAT4 was consistent with the previous analysis, and a significant causal relationship between IL-2 and STAT4 SNPs and osteoporosis was found by Mendelian randomization. Finally, through miRNA-mRNA network and drug sensitivity analysis, we analyzed to get Palbociclib/miR-141-3p/STAT4 axis, which can be used for the prevention and treatment of osteoporosis. In this study, we proposed the Palbociclib/miR-141-3p/STAT4 axis for the first time and provided new insights into the mechanism of oxidative stress in osteoporosis.

## Introduction

Osteoporosis is a common disease and a major public health problem worldwide, with more than 9 million osteoporosis-related fractures occurring each year, with associated morbidity and mortality^1^. Specifically, it refers to a systemic bone disease characterized by loss of bone mass, decreased bone strength, and deterioration of bone microstructure with age and aging of the human body^2^. Although the FDA has approved many drugs for the treatment of osteoporosis, most cases of osteoporosis remain untreated or ineffective due to the cost and side effects of currently available drugs^3^. Therefore, it is necessary to find new effective biomarkers and drugs for the prevention and treatment of osteoporosis.

Oxidative stress and inflammation have an impact on the pathobiology of almost all human diseases. More and more studies have reported that reactive oxygen species (ROS) may be an important cause of osteoporosis, and the oxidative stress induced by ROS plays an important role in osteoporosis^4^. ROS, which can be produced by both enzymatic and non-enzymatic systems, are central to the biology of oxidative stress. Under steady-state nonpathological conditions, low to moderate amounts of ROS play a major role in conventional cellular and mitochondrial signaling and function. However, if left unchecked, ROS may mediate oxidative tissue and cellular damage, leading to a cycle of inflammation and more oxidative stress^5^. It has been suggested that inflammation associated with oxidative stress and ageing promotes osteoporosis (mainly by reducing bone formation), excessive ROS can inhibit osteoblast bone formation and promote osteoclast bone resorption^6^. In addition, multiple human reports have demonstrated an inverse correlation between peripheral markers of oxidative stress and bone mass density in postmenopausal osteoporosis^7^. However, the biomarkers and molecular mechanisms of oxidative stress are still unclear.

Therefore, we collected multiple sequencing data of osteoporosis from GEO. Through differential analysis and WGCNA analysis, we obtained differentially expressed genes related to oxidative stress. Then, the correlation between hub genes and inflammatory factors, immune cell infiltration was further analyzed. Single-cell data analysis was also used to obtain the expression of hub genes in various cell subsets. In addition, we collected blood samples from hospitals for RT-qPCR and Elisa experiments to verify the hub genes, and analyzed the causal relationship between inflammatory cytokines, hub genes, and osteoporosis by Mendelian randomization. Finally, we constructed the miRNA-mRNA network and screened a new drug for the prevention and treatment of osteoporosis.

## Materials and methods

### Data acquisition, processing, and clinical sample collection

**Figure 1:**
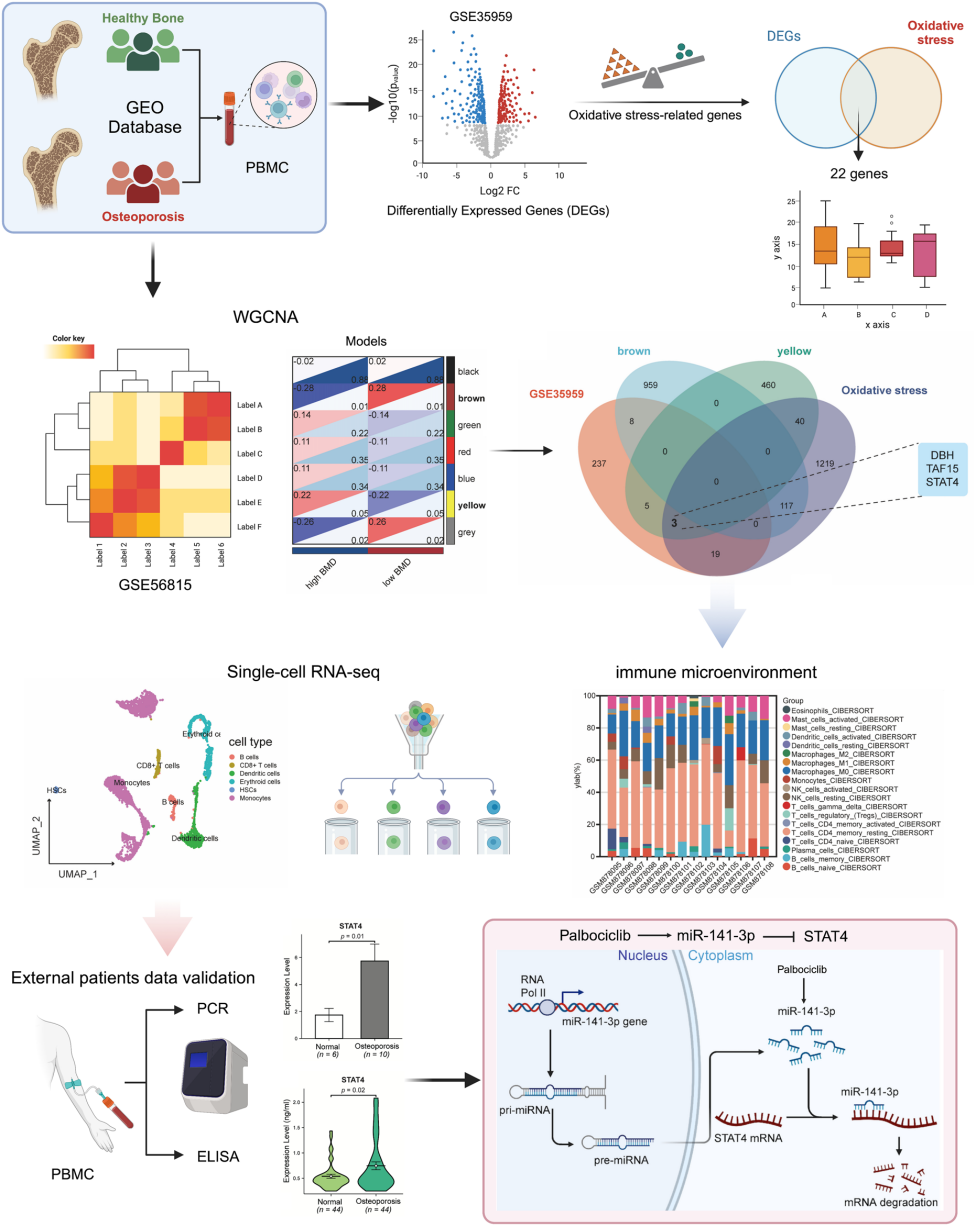
Flow chart of study design.

Figure 1 showed a flowchart of the study process. We obtained the gene expression datasets of normal individuals (N) and osteoporosis (OP) patients from the Gene Expression Omnibus (GEO) database (http://www.ncbi.nlm.nih.gov/geo/), Using the keyword “osteoporosis” to search for relevant gene expression datasets, excluding non-human samples. Including GSE35959 (N = 9, OP = 5) and GSE56815 (N = 40, OP = 40). In addition, miRNA-seq expression data was obtained from GSE159121 (N = 3, OP = 3), and Single-cell RNA-seq data was obtained from GSE147287^8^. We downloaded 1398 oxidative stress-related genes with a correlation score ≥ 7 From the GeneCards database (https://www.genecards.org) (Supplementary Table 1).

This study recruited 44 healthy individuals and 44 OP patients diagnosed with osteoporosis for Elisa; 6 healthy individuals and 10 OP patients for RT-qPCR. Inclusion criteria^9^: (1) age over 20 years old; (2) Bone Mineral Density (BMD) (Dual Energy X-ray Absorptiometry, DEXA) of the lumbar spine or total hip: T ≥  − 1.0 in the normal group and T ≤  − 2.5 in the OP group. Exclusion criteria were as follows: (1) any comorbidities (such as thyroid disease, diabetes, cancer, kidney disease, or ankylosing spondylitis) that might influence bone metabolism; (2) patients receiving Bisphosphonate, Teriparatide, calcitonin, etc. (3) Recent continuous bed rest for more than 3 months; (4) history of radiotherapy. The study was conducted in accordance with the Declaration of Helsinki and approved by the Institutional Ethics Committee of Xi’an Jiaotong University Honghui Hospital (No. 202308002).” for studies involving humans, and all patients signed informed consent.

### Differentially expressed genes related to oxidative stress between the OP group and the normal group

Firstly, the transcriptome data of PBMC from 9 OP patients and 5 normal subjects in GSE35959 were analyzed, data normalization and PCA analysis were performed, and the differential expression between the two groups was analyzed and displayed by volcano plot and heatmap (|Log_2_FC| > 1, p-value < 0.05). In addition, 272 differentially expressed genes (DEGs) were intersected with 1398 oxidative stress-related genes (OSRGs) and displayed by the Venn diagram. Finally, 22 differentially expressed genes related to oxidative stress associated with osteoporosis were obtained. In addition, differentially expressed miRNAs between OP and normal were identified by analyzing GSE159121 data.

### Consensus cluster and co-expression analysis

Using the gene expression profile of GSE56815, we calculated the MAD (Median Absolute) for each gene separately Deviation), the top 50% of the genes with the smallest MAD were excluded. The outlier genes and samples were removed by the R package WGCNA goodSamplesGenes method, and WGCNA was further used to construct a scale-free co-expression network.

β was a soft-thresholding parameter of 7. The sensitivity was set as 3, we also merged modules with less than 0.25 and finally obtained 7 co-expression modules. It is worth noting that the grey module is considered a set of genes that cannot be assigned to any module.

### GO and KEGG enrichment analysis of key module characteristic genes

By WGCNA analysis, we obtained the modules that were significantly associated with the phenotype and extracted the list of all genes within the module. Then, the “clusterProfile” package in R was used to perform Gene ontology (GO) and the Kyoto Encyclopedia of Genes and Genomes (KEGG)^10,11^ enrichment analysis of the genes in the module to clarify the potential pathogenesis and biological pathways of osteoporosis.

### Immune microenvironment analysis by CIBERSORT

CIBERSORT (http://cibersort.stanford.edu/), an online analytical platform based on a deconvolution algorithm, is used to assess the relevance of the correlation between TIICs in tissue and gene expression. Thus, CIBERSORT could be used to precisely estimate TIICs concentration in OP. The proportion of TIICs was visualized by the bar chart and the correlation heat map was drawn to detect the correlation between the 22 immune cells.

### Identification of hub genes and correlation analysis

Venn showed the intersection of WGCNA key module genes, oxidative stress-related genes, and osteoporosis differentially expressed genes, and three hub genes were obtained: DBH, TAF15, and STAT4. In addition, we analyzed the correlation between the three hub genes and inflammatory factors, as well as immune cell infiltration.

### Single-cell RNA‑seq analysis

Osteoporosis scRNA-seq data containing one patient was obtained from GSE147287 and analyzed with the “Seurat” package. To retain high-quality data, we eliminated cells with a mitochondrial gene percentage higher than 10%, fewer than three cell counts, fewer than 300 expressed genes, and more than 30,000 genes. For the identification of cell populations, we used the “Single R” package. The expression of hub genes in cell subsets was also analyzed. In addition, we used CellPhoneDB (an algorithm for analyzing intercellular communication at the single-cell level) to infer and quantify intercellular communication associated with subpopulations of cells in osteoporosis. Information flow was calculated for each signaling pathway, defined as inferring all communication probabilities between all cell pairs in the network. The communication between Dendritic cells and Monocytes is shown using a circle plot.

### Mendelian randomization (MR) analysis

We obtained GWAS data for 41 Cytokines from the website (https://data.bris.ac.uk/data/dataset/) and for an additional 22 Cytokines from IEU OpenGWAS. In addition, we extracted STAT4 SNPs from GWAS Catalog (https://www.ebi.ac.uk/gwas/), GWAS data for osteoporosis were obtained from the ukb-b-1214, and we performed Mendelian randomization analyses using the MR-Base platform, using the Inverse Variance Weighted (IVW) approach as our main analysis method, and MR Egger intercept was used to assess horizontal pleiotropy effects. We also performed a series of sensitivity analyses to test the robustness of causality, including Cochran’s Q test, MR Presso test, and leave-one-out analysis. The above statistical analyses were performed by “TwoSampleMR” (version 0.5.6) in R. p < 0.05 was considered significant unless otherwise stated.

### Real‑time quantitative real‑time polymerase chain reaction assays (RT‑qPCR)

FICOLL PAQUE PLUS (Cytiva, USA/Sweden) reagent was used to extract peripheral blood mononuclear macrophages from the subjects' peripheral blood whole blood. Then, RNA was extracted from peripheral blood mononuclear macrophages using the RNA fast200 total RNA extraction kit (Fastagen, Shanghai, China). The PrimeScript™ RT reagent Kit with gDNA Eraser RR047A Kit (TaKaRa, Japan) was used to remove genomic DNA first, and then reverse transcribed genomic DNA removed RNA into cDNA. Finally, the RT-qPCR was detected by using the Mjmimi real-time quantitative PCR instrument (Bio-Rad, USA) using the SYBR Green method. After the RT-qPCR reaction, the relative level of target genes was compared with GAPDH using the 2−ΔΔCt method.

### Enzyme-linked immunosorbent assay (ELISA)

Peripheral whole blood of subjects in the fasting state was extracted using a collection vessel containing separation glue. After collecting the required specimens, the samples were gently reversed and mixed 3–5 times immediately. After the blood was completely coagulated, the samples were left at room temperature for 3500 r/min, centrifuged for 5 min, and the upper serum was absorbed. Human Elisa Kit (YOBIBIO, Shanghai, China) was used to detect the concentration of target protein in the serum of the subjects, and the absorbance OD value at 450 nm was detected using the kit and the DENLEY DRAGON Wellscan MK 3 enzyme-labeled instrument (Thermo, Finland), and the concentration of standard substance was taken as the horizontal coordinate. The OD value is the ordinate coordinate to draw the standard curve, and finally, the target protein concentration is calculated according to the OD value of the sample.

### The network of miRNA–mRNA and drug prediction

The miRBase database (https://mirbase.org/) provides curated, comprehensive annotations of miRNA loci in a very large number of species. We obtained miRNAs targeting the STAT4 gene and constructed a miRNA–mRNA network, and the differential expression of miRNAs between the normal group and OP group was demonstrated by a heatmap. In addition, we use the TargetScan database (https://www.targetscan.org/) to show STAT4 and highly credible miRNA binding sites.

RNAactDrug database (http://bio-bigdata.hrbmu.edu.cn/RNAactDrug/) is a comprehensive resource for querying associations between drug sensitivity and RNA molecules including mRNAs, lncRNAs, and miRNAs at four molecular levels (expression, copy number variation, mutation, and methylation) from integrated analysis of three large-scale pharmacogenomic databases (GDSC, CellMiner, and CCLE)^12^. We obtained the correlation of miRNA, mRNA, and drug sensitivity. In addition, the 3D structure of the target miRNA was predicted by the RNAfold database (http://rna.tbi.univie.ac.at/cgi-bin/RNAWebSuite/RNAfold.cgi) and the Vfold3D database (http://rna.physics.missouri.edu/vfold3D/), and the drug molecular 3D structure was obtained by Pubchem.

### Statistical analysis

All raw data processing was conducted using R software (version 4.2.1). To detect significant differences between two independent groups, either a Student’s t-test or Wilcoxon’s rank sum test was employed. The Kruskal–Wallis test was used to examine differences when there were more than two independent groups. All p values were two-sided, with a statistical significance threshold assumed for p < 0.05.

### Ethical approval

The study was conducted in accordance with the Declaration of Helsinki and approved by the Institutional Ethics Committee of Xi’an Jiaotong University Honghui Hospital (No. 202308002)” for studies involving humans, and all patients signed informed consent.

## Results

### Differentially expressed genes related to oxidative stress

**Figure 2:**
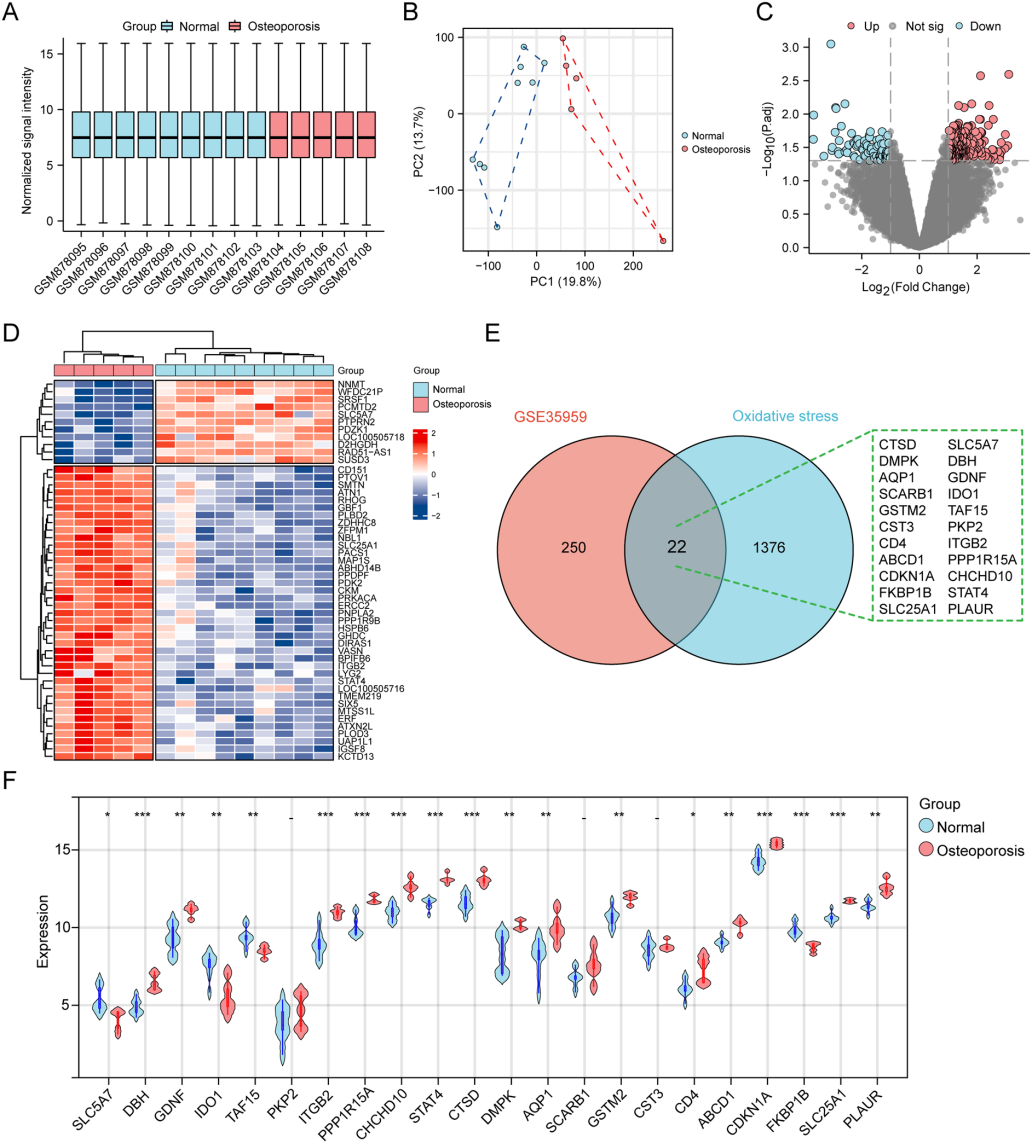
Differentially expressed genes related to oxidative stress between the OP and the normal groups. (**A**) Homogenization of the dataset samples. (**B**) PCA analysis of the dataset samples. (**C**) Volcano plot of differential gene analysis. (**D**) Heatmap plot of  the differential gene analysis. (**E**) The intersection of differential genes and oxidative stress related genes. (**F**) Violin plot of the expression of intersection genes in the two groups (***p  <  0.001, **p  <  0.01, *p  <  0.05).

We analyzed PBMC RNA-seq data from 9 normal individuals and 5 OP patients by GSE35959. Preliminary analysis of the data showed that the 14 samples were well homogeneous (Fig. 2A), and there was significant heterogeneity between the two groups (Fig. 2B). Therefore, we performed a differential expression analysis of the two groups, and the results obtained 272 differentially expressed genes, which were displayed by volcano plot (Fig. 2C) and heatmap (Fig. 2D). In addition, a list of 1398 oxidative stress-related genes was obtained by literature review, and the resulting 22 differentially expressed oxidative stress-related genes were shown by the Venn diagram: SLC5A7, DBH, GDNF, IDO1, TAF15, PKP2, ITGB2, PPP1R15A, CHCHD10, STAT4, CTSD, DMPK, AQP1, SCARB1, GSTM2, CST3, CD4, ABCD1, CDKN1A, FKBP1B, SLC25A1, and PLAUR (Fig. 2E). Violin plots were used to show the expression of these genes in the two groups (Fig. 2F).

### Identification of key modules by WGCNA

**Figure 3:**
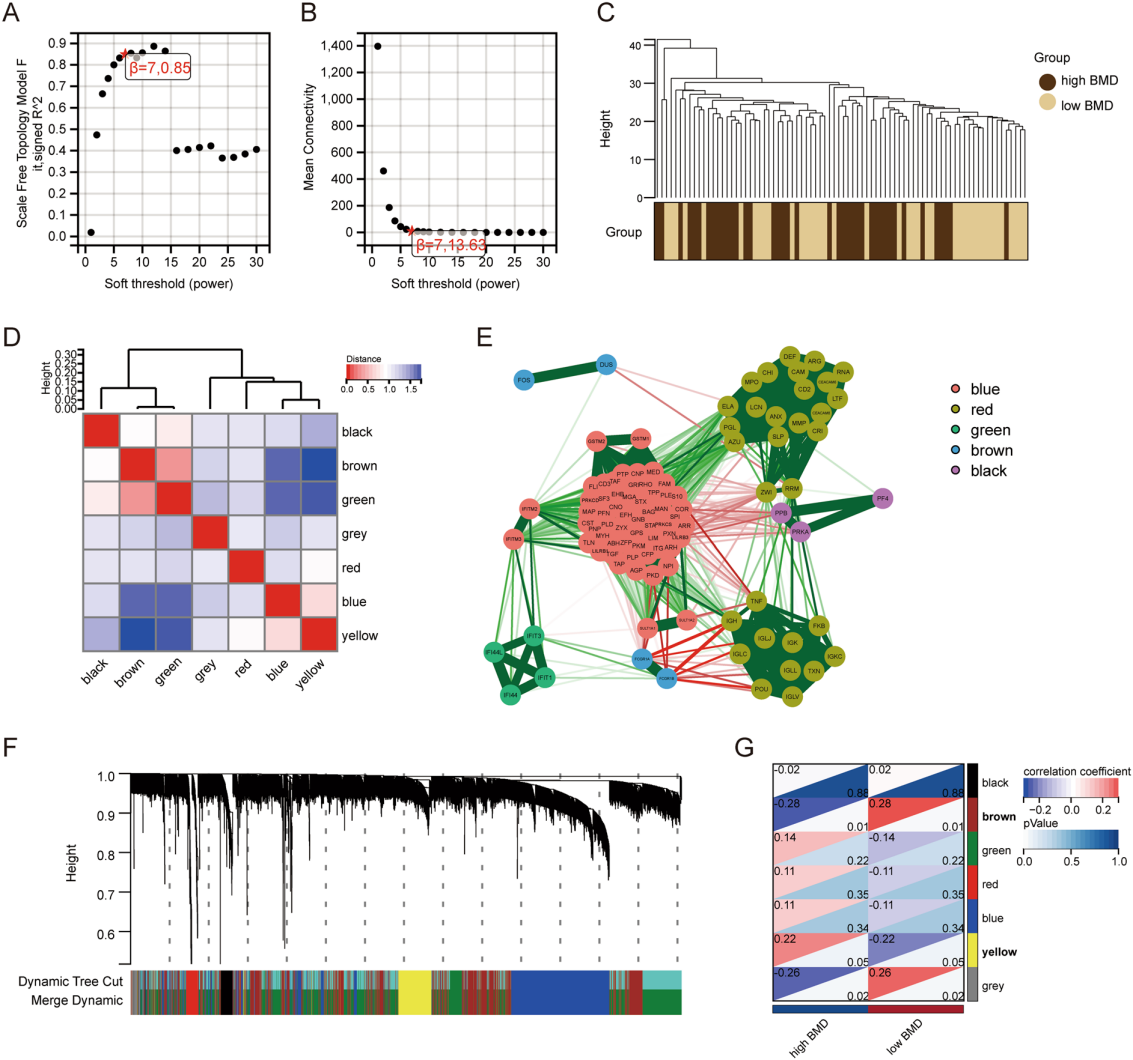
Consensus cluster and co-expression analysis (WGCNA). (**A**) Scale independence in the case of different soft thresholds, scale-free fit index (Y-axis) for different soft thresholds (X-axis). (**B**) Average connectivity for different soft thresholds. (**C**) A cluster tree of gene samples drawn by the co-expression network was constructed based on the optimal soft threshold. (**D**) Correlation heatmap between the seven cluster modules. (**E**) Gene co-expression in different modules. (**F**) Cluster trees of genes drawn after partitioning genes into different modules. (**G**) Correlation heatmap showing the correlation and significance between modules and clinical features of interest.

We performed WGCNA analysis on GSE56815 (N = 40, OP = 40) data and set the best soft-thresholding parameter β to 7 for subsequent analysis (Fig. 3A,B). The cluster diagram showed the clustering between the two groups (Fig. 3C) and the 7 modules corresponding to the branches (Fig. 3F). The correlation plot showed the correlation between the 7 modules, and the color indicates the distance (Fig. 3D). Figure 3E showed the co-expression between module genes, with thicker lines indicating stronger co-expression relationships. Finally, the correlations between 7 modules and phenotypes were obtained, among which the yellow (p = 0.05) and brown (p = 0.01) modules were significantly correlated with the level of bone mineral density (BMD) (Fig. 3G), we will conduct subsequent analyses of these two modules.

### GO/KEGG enrichment analysis of brown and yellow modules

**Figure 4:**
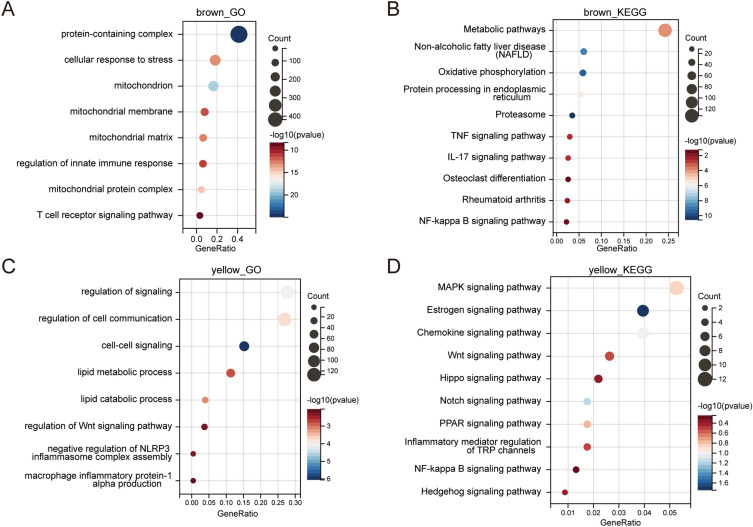
GO/KEGG enrichment analysis of genes in the brown and yellow modules. (**A**) GO enrichment analysis of genes in the brown module. (**B**) KEGG enrichment analysis of genes in the brown module. (**C**) GO enrichment analysis of genes in the yellow module. (**D**) KEGG enrichment analysis of genes in the yellow module.

We performed GO/KEGG functional enrichment of 1084 genes in the brown module and 508 genes in the yellow module obtained by WGCNA. Specifically, GO analysis of brown module genes showed that they were related to various cellular functions, such as protein-containing complex, cellular response to stress, mitochondrion, and regulation of innate immune response (Fig. 4A). KEGG analysis showed that it was related to a variety of pathways, such as Metabolic pathways, Oxidative phosphorylation, TNF signaling pathway, IL-17 signaling pathway, and Osteoclast differentiation (Fig. 4B). In addition, GO analysis of yellow module genes showed that they were related to a variety of cellular functions, such as regulation of cell communication, cell–cell signaling, lipid metabolic process, and negative regulation of NLRP3 inflammasome complex assembly (Fig. 4C). KEGG analysis showed that it was related to a variety of pathways, such as MAPK signaling pathway, Estrogen signaling pathway, Chemokine signaling pathway, Wnt signaling pathway, Notch signaling pathway, PPAR signaling pathway, Inflammatory mediator regulation of TRP channels, NF-kappa B signaling pathway, and Hedgehog signaling pathway (Fig. 4D).

### Identification of hub genes, correlation analysis of cytokines, and Mendelian randomization analysis

**Figure 5:**
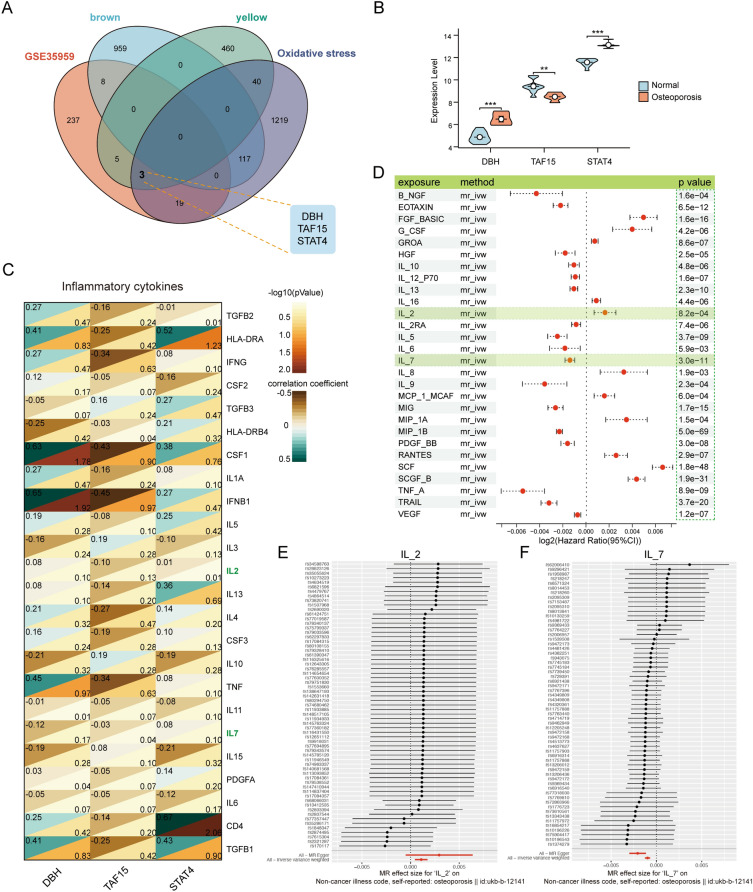
Identification of hub genes, correlation analysis of cytokines, and Mendelian randomization analysis. (**A**) The Venn diagram showing the screening of the three hub genes. (**B**) Violin plot of the expression of the three hub genes in the two groups. (**C**) Heat map of the correlation between the three hub genes and inflammatory factors, with correlation coefficient in the upper left corner and *p*-value in the lower right corner. (**D**) Forest plot of the Mendelian randomization analysis between cytokines and osteoporosis. (**E**) Forest plot of Mendelian randomization analysis between IL-2 and osteoporosis. (**F**) Forest plot of Mendelian randomization analysis between IL-7 and osteoporosis (***p < 0.001, **p < 0.01, *p < 0.05).

We obtained three hub genes by Venn diagram analysis of OP differentially expressed genes, oxidative stress-related genes, and yellow module genes, which were DBH, TAF15, and STAT4 (Fig. 5A). The box plot shows the expression of the three genes in the two groups, which shows that DBH and STAT4 are significantly higher in the OP group, while TAF15 is significantly lower in the OP group (Fig. 5B).

The correlation between the three genes and inflammatory cytokines was analyzed and shown by heatmap (Fig. 5C). The results showed that DBH was significantly negatively correlated with IL-11 (cor =  − 0.01, p = 0.01); TAF15 was negatively correlated with HLA-DRB4 (cor =  − 0.03, p = 0.04) and IL-7 (cor =  − 0.03, p = 0.04). STAT4 has a significant negative correlation with TGFB2 (cor =  − 0.01, p = 0.01) and a significant positive correlation with IL-2 (cor = 0.01, p = 0.01). In addition, we used Mendelian randomization to analyze the data from GWAS, Fig. 5D shows the causal relationship between multiple cytokines and osteoporosis through forest plot, and the results show that IL-2 as a risk factor has a significant causal relationship with osteoporosis, while IL-7 as a protective factor has a significant causal relationship with osteoporosis. Specifically, the total MR Effect size between SNPs of IL-2 (Fig. 5E), IL-7(Fig. 5F), and osteoporosis were presented by forest plots, the IVW method was used as the main analysis method.

### Immune infiltration and correlation analysis

**Figure 6:**
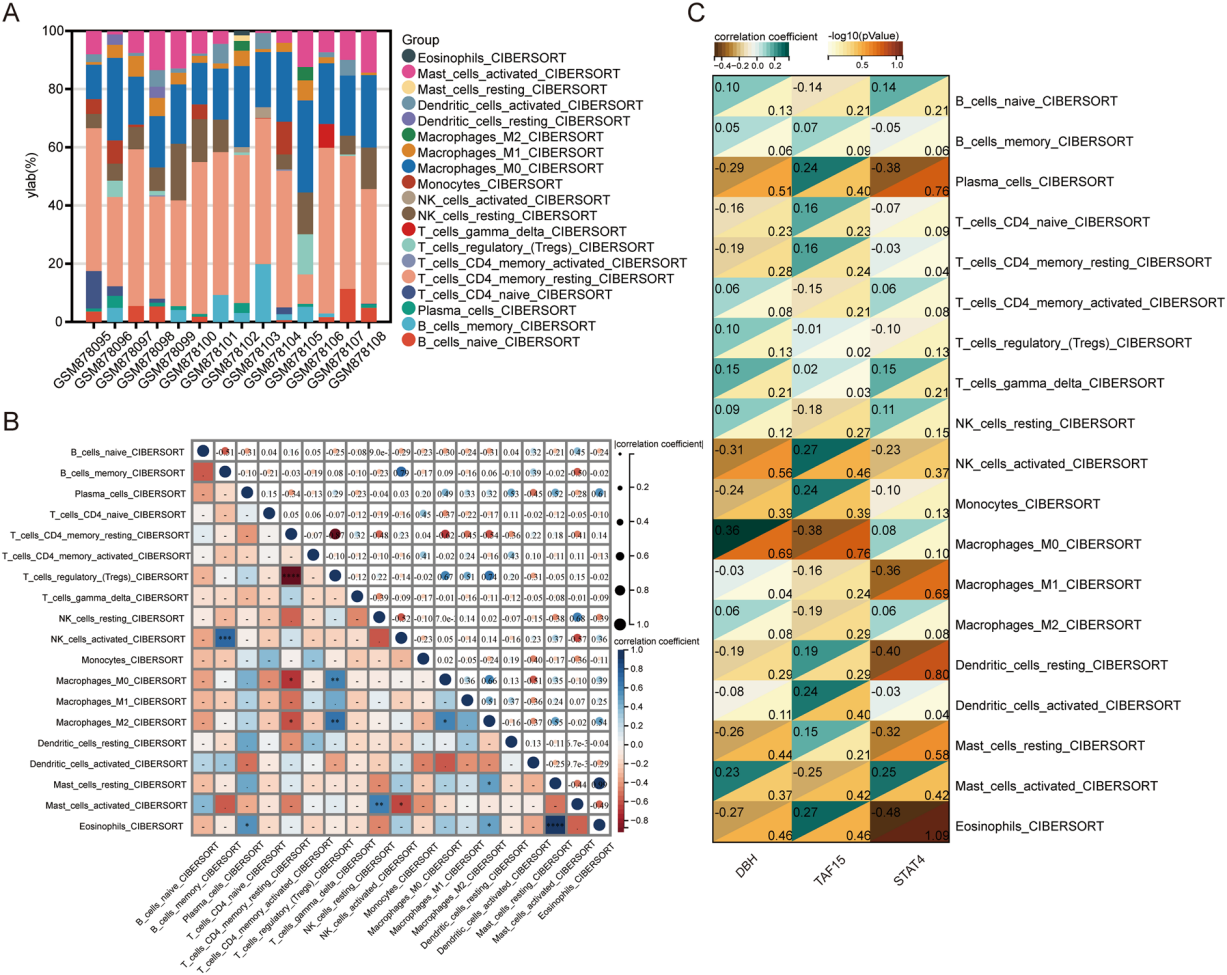
Immune infiltration and correlation analysis. (**A**) The stacked plot of the proportion of immune cell infiltration. (**B**) Heatmap of the correlation between immune cell infiltration, blue indicates positive correlation and red indicates negative correlation. (**C**) Heatmap of the correlation between the 3 hub genes and immune cell infiltration (***p  <  0.001, **p  <  0.01, *p  <  0.05).

The infiltration of immune cells in 14 samples was obtained by CIBERSORT analysis, the proportion of immune cells was displayed by stacking plot (Fig. 6A), and the correlation between immune cells was displayed by heat map (Fig. 6B). Specifically, it was found that the main constituent immune cells were M0 Macrophages and CD4 memory-resting T cells. M0 Macrophages (cor =  − 0.63, p < 0.05), M2 Macrophages (cor =  − 0.54, p < 0.05) and CD4 memory-resting T cells have a significant negative correlation. M0 Macrophages (cor = 0.67, p < 0.01), M2 Macrophages (cor = 0.74, p < 0.01), and Regulatory T cells (Tregs) have a significant positive correlation. In addition, we demonstrated the correlation between three hub genes and immune cell infiltration by heatmap (Fig. 6C). The results showed that DBH was negatively correlated with M1 Macrophages (cor =  − 0.03, p = 0.04). TAF15 was positively correlated with Gamma delta T cells (cor = 0.02, p = 0.03) and negatively correlated with Tregs (cor =  − 0.01, p = 0.02). STAT4 was negatively correlated with CD4 memory-resting T cells (cor =  − 0.03, p = 0.04) and activated Dendritic cells (DCs, cor =  − 0.03, p = 0.04).

### Single-cell RNA‑seq analysis

**Figure 7:**
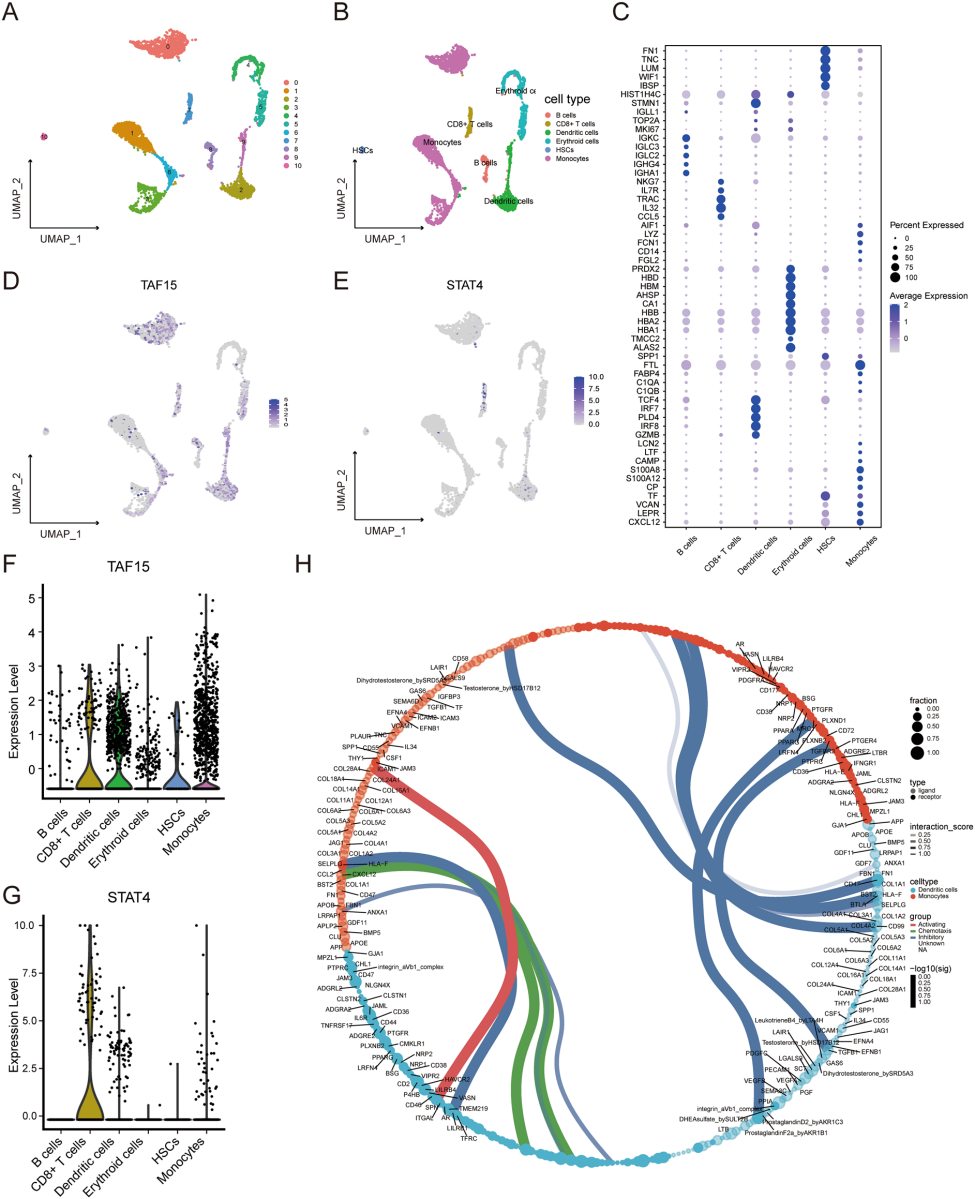
Single-cell analysis. (**A**) Cluster map of cell subsets. (**B**) Cluster map after naming the cell subsets. (**C**) The dot plot shows the expression of markers in different cell subsets. (**D**) TAF15 gene expression in clusters of different cell subsets. (**E**) STAT4 gene expression in clusters of different cell subsets. (**F**) The violin plot shows the expression level of the TAF15 gene in different cell subsets. (**G**) The violin plot shows the expression level of the STAT4 gene in different cell subsets. (**H**) Circle diagram of signaling communication between DCs and monocytes.

We analyzed a single-cell RNA-seq sample of osteoporotic from the GSE147287 and clustered the cells into 5 cell subsets, namely: B cells, CD8+ T cells, Dendritic cells, Erythroid cells, HSCs, and Monocytes (Fig. 7A,B). The markers of each subset are shown in a dot plot (Fig. 7C), and the expression of the TAF15 and STAT4 genes in each subset is shown in violin plots (Fig. 7D–G). The results showed that TAF15 was mainly expressed in Dendritic cells and Monocytes and STAT4 was mainly expressed in CD8+ T cells, Dendritic cells, and Monocytes. We chose monocytes to further subdivide the subsets and the markers have been summarised in Supplementary Table 3. A total of three subsets were included: Classical monocytes, Intermediate monocytes, and Non-classical monocytes (Supplementary Fig. 1A). In addition, we demonstrated the expression of genes TAF15 and STAT4 in the three subsets (Supplementary Fig. 1B,C) and found that TAF15 was expressed in all three monocyte subsets, whereas STAT4 was expressed only in small amounts in Intermediate monocytes. In addition, the interaction between Dendritic cells and Monocytes was analyzed using the “CellPhoneDB” package and shown in a circle diagram (Fig. 7H). The results showed that the interaction between two cells was activated through ICAM1-SPN ligand receptors.

### Experimental validation and Mendelian randomization

**Figure 8:**
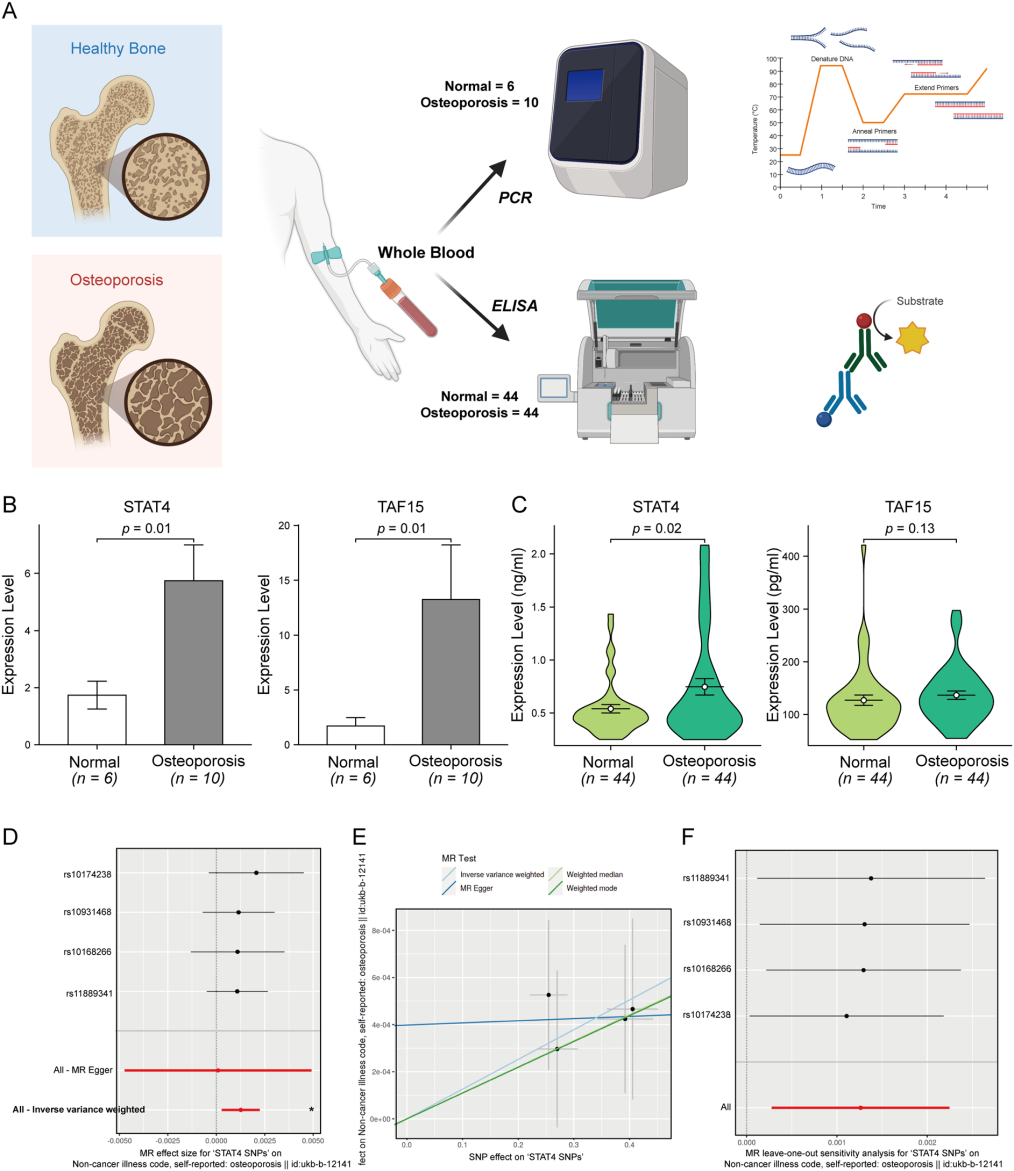
Experimental validation and Mendelian randomization. (**A**) The schematic diagram of the RT-qPCR and Elisa experiments. (**B**) The expression levels of STAT4 and TAF15 genes in the two groups were obtained by RT-qPCR. (**C**) The expression levels of STAT4 and TAF15 genes in the two groups were obtained by Elisa. (**D**) Forest plot of the total effect size of STAT4 SNPs on osteoporosis outcome. (**E**) Scatter plot of Mendelian randomization between STAT4 SNPs and osteoporosis outcome. (**F**) Leave-one-out analysis of the remaining stability after stepwise elimination of the SNP.

We collected blood samples from 6 normal people and 10 osteoporosis patients in the ward for RT-qPCR, and 44 normal people and 44 osteoporosis patients for Elisa. Figure 8A shows the experimental design. Figure 8B shows the RT-qPCR results, where both STAT4 and TAF15 were significantly highly expressed in the OP group. Figure 8C shows the Elisa results, where STAT4 was also significantly highly expressed in the OP group, while TAF15 was not differentially expressed. Of interest, the experimental results for STAT4 were consistent with the RNA-seq results. Mendelian randomization analysis found that there was a causal relationship between STAT4 as a risk factor and osteoporosis in general (p < 0.05, Fig. 8D). The scatter plot also showed that the correlation is positive under various analysis methods (Fig. 8E). In addition, Leave-one-out analysis showed that the remaining SNPs were still relatively stable after stepwise elimination of SNPs (Fig. 8F).

### miRNA–mRNA network and drug prediction

**Figure 9:**
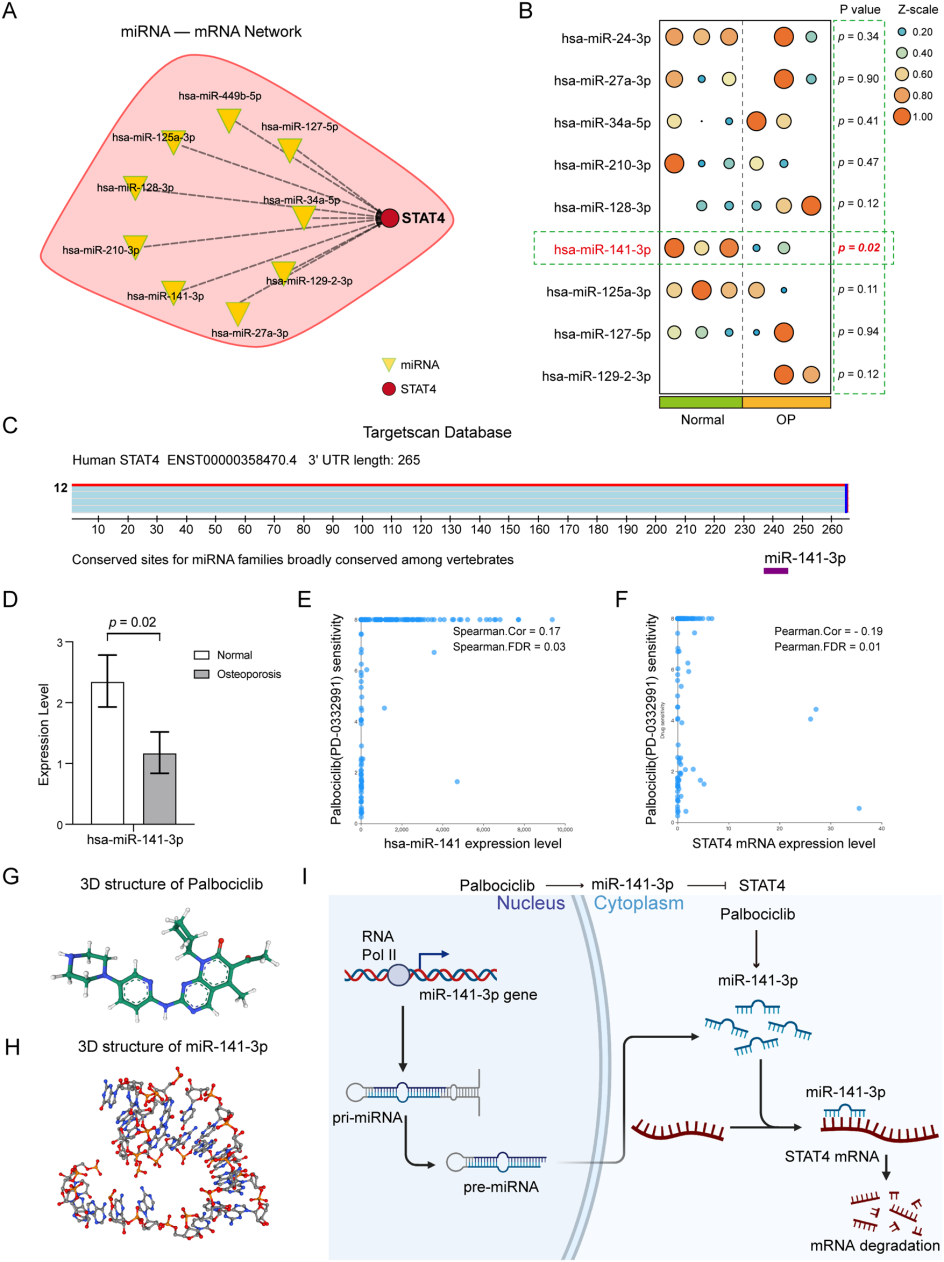
miRNA-mRNA network and drug prediction. (**A**) miRNA-STAT4 mRNA network. (**B**) Differential expression of miRNAs in the two groups. (**C**) The conserved binding site of miR-141-3p to STAT4 mRNA. (**D**) The expression of miR-141-3p was significantly lower in the OP group. (**E**) Correlation between miR-141 expression level and Palbociclib drug sensitivity (Cor = 0.17, FDR = 0.03). (**F**) Correlation between STAT4 mRNA expression level and Palbociclib drug sensitivity (Cor =  − 0.19, FDR = 0.01). (**G**) 3D structure of Palbociclib. (**H**) Predicted 3D structure of miR-141-3p. (**I**) The mechanistic diagram of the Palbociclib/miR-141-3p/STAT4 axis.

We summarized and plotted the network of miRNAs targeting STAT4 through the miRbase database (Fig. 9A), obtained the expression of miRNAs in normal and OP groups using miRNA sequencing data analysis (Fig. 9B) (Supplementary Table 2), and found that only miR-141-3p was significantly different (p = 0.02), which was lowly expressed in OP group (Fig. 9D). In addition, the Conserved sites between miR-141-3p and STAT4 mRNA were obtained from the TargetScan database (Fig. 9C). RNAactDrug database analysis showed that Palbociclib (PD-0332991) sensitivity was significantly positively correlated with miR-141 expression level (Fig. 9E, Cor = 0.17, FDR = 0.03) and significantly negatively correlated with STAT4 mRNA expression level (Fig. 9F, Cor =  − 0.19, FDR = 0.01). Furthermore, the 3D structure of Palbociclib was obtained by the PubChem database (Fig. 9G), and the 3D structure of miR-141-3p was predicted by RNAfold and Vfold3D databases (Fig. 9H). Finally, we plotted the molecular mechanism (Fig. 9I).

## Discussions

Osteoporosis is the most common bone disease, and it is estimated that more than 200 million people suffer from it worldwide^13^. And it is increasing due to the ageing of the population worldwide, resulting in an increasing number of postmenopausal women with osteoporosis^14^. It is likely that the pathology of osteoporosis is significantly influenced by oxidative stress and the over-activation of osteoclasts caused by reactive oxygen species (ROS)^4^. It has been suggested that oxidative stress is an independent risk factor for osteoporosis and that it may interfere with the osteogenic differentiation of mesenchymal stem cells (MSCs), making it an important factor in the development of the disease^15^. However, the biomarkers and molecular mechanisms of oxidative stress in osteoporosis remain obscure.

Therefore, we collected the miRNA, mRNA, and single-cell OP data, and by differential, WGCNA clustering and Venn screening analysis, three differentially expressed genes related to oxidative stress were obtained: DBH, TAF15, and STAT4. Then, the correlation between hub genes and inflammatory factors, immune cell infiltration was further analyzed. Single-cell data analysis was also used to obtain the expression of hub genes in various cell subsets. In addition, we collected human blood samples from the hospital for RT-qPCR and Elisa experiments to validate hub genes, and analyzed the causal relationship between inflammatory cytokines, STAT4, and osteoporosis by Mendelian randomization. Finally, we constructed a network to obtain the miR-141-3p/STAT4 axis and obtained a new drug Palbociclib for the prevention and treatment of osteoporosis through drug sensitivity analysis.

In this study, WGCNA analysis revealed two significant gene modules in brown and yellow. The genes within the yellow module were significantly associated with intercellular signal transduction, negative regulation of NLRP3 inflammasome assembly, MAPK signaling pathway, and chemokine signaling pathway. A previous study had shown that high expression of NLRP3 and IL-1β inhibited osteogenic differentiation, thereby triggering the development of osteoporosis^16^. Therefore, the progression of the disease can be inhibited by suppressing the activity of NLRP3 inflammasomes and the expression of IL-1β. This is consistent with the function of the yellow module genes, which negatively regulate NLRP3 inflammasome assembly.

Recent osteoimmunological studies have shown that the immune system and immune factors play a crucial regulatory role in the development of osteoporosis, emphasizing the need for greater awareness of the link between immunity and osteoporosis. Aberrant activation of the immune system upsets the balance between osteoblasts and osteoclasts, causing an imbalance in bone remodeling and leading to osteoporosis^17^. Monocytes and the pathophysiology of osteoporosis have been studied for several years^18^. However, monocyte subpopulation identification in osteoporosis has been poorly reported. Intermediate monocytes are thought to be recruited to sites of inflammation and have a pro-inflammatory effect, like classical monocytes. An increase in intermediate monocytes has been associated with several human inflammatory diseases, including sepsis, rheumatoid arthritis, tuberculosis, HIV and congestive heart failure. Frucht et al. demonstrated previously unrecognized expression of STAT4 in monocyte lineage cells. They hypothesized that STAT4 expression reflects the point of differentiation of human monocytes. IFN-α signaling through STAT4 is a transduction pathway common to both lymphocytes and monocytes and complements IL-12 signaling when IL-12 or its receptor is not available^19^. Our results also found that STAT4 was highly expressed in a small number of Intermediate monocytes, which may be limited to the overall sample and cell number.

Some studies have shown that senile osteoporosis can be treated by preventing macrophages from producing osteoclasts, but the exact mechanism of macrophages in osteoporosis remains to be investigated^20^. We analyzed the correlation between hub genes and immune cell infiltration and found that DBH was significantly negatively correlated with M1 macrophages. M1 or classical activation of macrophages is an important inflammatory response. Polarized M1 can produce high levels of reactive oxygen species (ROS), nitric oxide (NO), and proinflammatory cytokines such as interleukins IL-1, IL-2, IL-6, IL-12, TNF-α, and IFN-γ^21^. Interestingly, we found a significant positive correlation between STAT4 and IL2. At the same time, the MR results showed that the increase of IL-2 could lead to the occurrence of osteoporosis, and there was a significant causal relationship between them. In addition, some studies have shown that the loss of dendritic cells causes local immune environment disruption and destruction of immune function, suggesting that dendritic cells are closely related to inflammatory bone loss^22^. This may be related to the presentation of dendritic cells to activate T cells, thereby indirectly playing a role in immune-mediated inflammatory bone loss. It is likely that dendritic cell-derived osteoclasts show enhanced bone resorption activity and the expression of osteoclast-related molecules is also increased^23^. Meanwhile, our results also showed a significant negative correlation between STAT and activated DCs, suggesting that STAT4 may promote the development of osteoporosis through the loss of DCs.

Verified by RT-qPCR and Elisa experiments, we found that the STAT4 gene was significantly up-regulated in the osteoporosis group. Signal transducers and activators of transcription (STATs) modulate transcription by signaling from activated cell surface receptors to the nucleus and are key components of various signaling pathways. The mammalian STAT family comprises seven members, STAT1, STAT2, STAT3, STAT4, STAT5a, STAT5b and STAT6, all encoded by different genes^24^. STAT4 is a key mediator of inflammation and tumor development. Understanding the molecular mechanisms of STAT4 in immune responses and immune-mediated diseases will help to develop new therapeutic options for human diseases. Different combinations of STAT4 can be activated by a variety of cytokines, including interleukin (IL)12, type I interferon (IFN-I), IL-23, IL-2, IL-27, IL-35, etc^25^. MiRNAs act by degrading mRNA and regulating post-transcriptional modifications of gene expression, thereby repressing protein translation. miRNA-132, miRNA-212, and miRNA-200a down-regulate STAT4 in NK cells by targeting STAT4 3ʹ-UTR^26^. To further explore the upstream molecular mechanism of STAT4, we constructed a miRNA-mRNA network and combined it with the miRNA-seq result, we found that miR-141-3p was significantly down-regulated in the OP group, which was consistent with the high expression of STAT4 in the OP group, and there was a stable binding site with STAT4 mRNA. MiR-141-3p not only restricted the migration and invasion of gastric cancer cells, but also inhibited the transformation of normal fibroblasts (NFs) into cancer-associated fibroblasts (CAFs) by targeting the STAT4/WNT/β-catenin pathway. Meanwhile, the Dual-luciferase report assay was employed to determine the direct binding of miR-141 to the STAT4 3ʹ UTR^27^. Ma et al. confirmed STAT4 as a direct target gene of miR-141; miR-141 inhibited the growth and metastasis of liver cancer cells through the targeting of STAT4^28^. In addition, Recep Bayraktar et al. suggest that miR-141-3p reduced the inflammatory response by inhibiting signal transducer and activator of transcription 4 (STAT4) in mice with EAM^29^. Thus, the low expression of miR-141-3p in OP might promote the inflammatory response, this is the first reported relationship between miR-141-3p and STAT4 in osteoporosis. In addition, the results of drug sensitivity analysis showed that there was a significant positive correlation between Palbociclib and miR-141 expression, but a significant negative correlation between Palbociclib and STAT4 expression, indicating that Palbociclib may mediate the degradation of STAT4 mRNA by acting on miR-141 to inhibit the development of osteoporosis. Palbociclib has recently been approved for the treatment of advanced breast cancer, targeting CDK4 and CDK6^30^. One of the most common long-term effects of breast cancer treatment is osteoporosis, with up to 80% of breast cancer patients experiencing bone loss^31^. Therefore, our results suggest that the use of Palbociclib drug may be of benefit to breast cancer patients in terms of the reduction of the risk of bone loss.

Although our study proposed the Palbociclib/miR-141-3p/STAT4 axis for the first time and provided new insight into the mechanism of oxidative stress in osteoporosis, there are limitations that need to be considered. First, the sample size was small, and more data sources needed to be integrated. Second, self-test samples are necessary for further verification of the analysis results. Third, the expression situation is only a preliminary verification, and further experimental exploration of the mechanism will be necessary. In addition, Yau et al.^32^ have shown that genetic factors may contribute to differences in BMD within and between races. Thus, differences in genetic structure between populations need to be considered when predicting, diagnosing, intervening, and predicting risk for osteoporosis.

## Conclusions

In conclusion, our study proposed the Palbociclib/miR-141-3p/STAT4 axis for the first time and provided a new perspective on the mechanism of oxidative stress in osteoporosis. Our results confirmed that STAT4 may be a novel biomarker for the prevention and treatment of osteoporosis. However, further research is needed to understand the molecular mechanism by which STAT4 regulates the development of osteoporosis.

### Supplementary Information

Supplementary Information.

## Data Availability

Data sources can be found in the “Materials and methods” section, from the Gene Expression Omnibus (GEO) database (http://www.ncbi.nlm.nih.gov/geo/). Including GSE35959 (N = 9, OP = 5) and GSE56815 (N = 40, OP = 40); MiRNA-seq data was obtained from GSE159121 (N = 3, OP = 3), and Single-cell RNA-seq data was obtained from GSE147287. Other materials such as codes can be obtained by contacting the Corresponding Author.

## Abbreviations

GEO: The Gene Expression Omnibus database
WGCNA: Weighted correlation network analysis
BMD: Bone mineral density
DEXA: Dual energy X-ray absorptiometry
MAD: Median absolute
GO: Gene Ontology
KEGG: The Kyoto Encyclopedia of Genes and Genomes
IVW: Inverse variance weighted
RT‑qPCR: Real‑time quantitative real‑time polymerase chain reaction assays
ELISA: Enzyme-linked immunosorbent assay
MR: Mendelian randomization
ROS: Reactive oxygen species
NO: Nitric oxide
Tregs: Regulatory T cells
DCs: Dendritic cells
MSCs: Mesenchymal stem cells
STATs: Signal transducers and activators of transcription
IL: Interleukin
IFN-I: Type I interferon
